# The Hospital Nursing Supplement

**Published:** 1896-11-21

**Authors:** 


### "The Hospital" Nursing Mirror.

BEING THE EXTRA NURSING SUPPLEMENT OF "THE HOSPITAL."

[Contributions for this Supplement should be addressed to the Editor, THE HOSPITAL, 28 & 29, Southampton Street, Strand, London, W.C., and should have the word "Nursing" plainly written in left-hand top corner of the envelope.]

### News from the Nursing World.

### "THE LONGEST REIGN" AND THE QUEEN'S NURSES.

THE Queen Victoria Jubilee Institute for Nurses seems likely to profit exceedingly, and deservedly, in the year that is coming—the year which beats the record in reigns for England. The Primrose League is proposing to raise funds in its behalf, and a letter which appeared in the London daily papers last week over a number of influential names, urges by way of suitable commemoration the raising of a large sum, "to increase in magnitude and endow in perpetuity" this great work for the poor, "of which the Queen herself laid the foundation, and to which she gave £70,000 in the Jubilee year." District nurses are being increasingly and rightly looked upon as the most important educational influence at work amongst the poor at large. All who recognise this will feel that the universal spread of trained nursing amongst the poor in their own homes is the best of all ways to better their condition. Money only is needed in order to extend this good work "to every town and district in the United Kingdom," and towards this object, which is sure to command the Queen's personal approval and sympathy, help is invited, and will surely be forthcoming. A meeting will shortly be called to appoint an executive committee to carry out the scheme. In the meantime communications should be addressed to the hon. secretaries of "The Queen's Commemoration Fund," at 64, Cannon Street, E.C.

### KING'S COLLEGE HOSPITAL GUILD.

AN excellent plan has been started at King's College Hospital to provide clothing and other comforts for the patients while in the hospital, the expense of the hundred and one little necessaries wanted in the wards too often falling upon the sisters, who now find most of the indispensable flannel shirts, children's frocks, &c., from their own resources. By means of a guild composed of many members this difficulty will be met, the burden removed from the sisters' shoulders, and much advantage result to the patients. The guild is to consist of presidents, vice-presidents, working members, and associates, each president finding three vice-presidents, who in their turn find at least five working members, and as many associates as possible, the latter sending subscriptions and contributions in kind to their vice-presidents each year before October 20th, and the vice-presidents sending on all together to their presidents before November 1st, to forward to the hon. secretary at once. Two articles of clothing and one shilling subscription is expected each year from working members, and a five shilling subscription from associates. The privilege of belonging to the guild is not to be confined to the feminine element, but it is hoped that men associates will be plentiful, and contributions from them in money and substantial "comforts," wheeled chairs, bed-rests, and other useful gifts, well to the fore. Lists of the special garments required from working members, patterns, and full particulars, may be obtained from the Hon. Secretary, Miss Plunket, "Somers," Wimbledon, S.W.

### CHEYNE HOSPITAL FOR CHILDREN.

THE annexe which it is proposed to add to the Hospital for Sick and Incurable Children, Cheyne Walk, and in aid of which a sale of needlework is to be held at the hospital on Wednesday and Thursday, November 25th and 26th, is necessitated by the present want of room for the nursing staff. The number of nurses was increased three years ago, putting a heavy strain on the existing sleeping accommodation. Besides this it is desired to provide a larger isolation ward. The proposed addition will be erected in Lawrence Street, where a site has already been secured, connected with the main building by a covered way. Plans are not yet completed, but the probable cost is estimated at from £1,200 to £1,400. Donations towards the "Lawrence Street Annexe Fund" will be gratefully received by the Secretary at the hospital.

### CARDIFF INFIRMARY BAZAAR.

LADY WINDSOR and a band of willing helpers have been long working hard to ensure the success of the great bazaar in aid of the Cardiff Infirmary, which took place last week, and their efforts were certainly crowned with triumph. Lord Windsor, who has been Mayor of Cardiff for the past year, opened the proceedings in state at the Drill Hall, which was made beautiful for the occasion with effective decorations. The stalls were one and all very attractively got up; that in charge of the "infirmary nurses," presided over by Miss Mont-Wilson, was, of course, specially popular, and did brisk business. The total proceeds of the first day, including purses presented to Lady Windsor, reached £1,633. It is hoped that this bazaar will realise some £4,000, and help largely towards clearing off the present debt on the infirmary, which, it will be remembered, has been greatly added to and improved, particularly in the way of accommodation for the nursing staff, and enabling the committee to open those new wards which are not yet in use for lack of funds.

### PETER BROUGH DISTRICT NURSING ASSOCIATION, PAISLEY.

THE fourth annual report of the Paisley (Peter Brough) District Nursing Association, which is affiliated with the Queen's Jubilee Institute, is an entirely satisfactory one, and chronicles, amongst other pleasant things, the progress of a new home now being built in Oakshaw street to accommodate the superintendent and nine nurses, fitted with all modern conveniences and comforts. This home will be the first in Scotland specially built for Queen's nurses. The association was visited by Miss Wade, superintendent of the Edinburgh Central Home, early in the present year, and by Miss Peter in

September, in both instances much praise being given to the work of the nurses and the general organisation of the home under the care of Miss Watson, its superintendent, recently a recipient of the silver badge. The report contains a short account of the never-to-be-forgotten visit to Windsor in July, at which Miss Watson was present with her staff. It is found that the doctors are availing themselves more and more of the services of the nurses, whose help in acute cases and at operations, as well as for a large number of "chronics," is gratefully appreciated.

### CARNARVON DISTRICT NURSE SOCIETY.

The committee of the Carnarvon District Nurse Society are proposing to hold a "café chantant" and sale of plain and fancy articles in the Carnarvon Guild Hall on December 3rd and 4th, and invite all "who sympathise with the valuable work Nurse Davies (Queen's Nurse) is doing" to show their interest by sending contributions towards furnishing the stalls. Miss Pugh, Bryn Menai, is acting as hon. secretary. Funds to carry on the work of the society amongst the sick poor of the district are very much needed.

### GLASGOW CANCER HOSPITAL.

The patients in the Glasgow Cancer Hospital have just been moved into the new building which has been preparing in Hill Street. The new hospital has been constructed out of two existing buildings with a connecting addition, and now contains thirty-two beds. It is fitted throughout with electric light. The nurses' quarters have been comfortably planned, a portion of the building being set apart as a home. The old hospital, it is expected, will be used as a "Home of Peace" for cases in the future.

### UP-COUNTRY NURSING ASSOCIATION IN INDIA.

The first report of this association is interesting reading, and is evidence that, so far, the experiment, which had for its object the supply of English trained nurses to Europeans in up-country districts in India, has been successful. The first nurse was sent to the North-West Provinces in May, 1894, a second and third followed in September of that year, and these were received into the Nursing Home attached to the Ramsay Hospital at Nyme Tal, their services being speedily and continuously claimed. In 1895 a nurse was sent to Quetta, and two more have since gone to the North-West Provinces. An attempt to organise a branch in the Punjab was not successful. The work of the nurses has been much appreciated, and the committee trust that other provinces will in time establish branches. The inestimable blessing of skilled nursing in those out-of-the-way places in the hills, where the private nurse working on her own account does not usually penetrate, can, perhaps, be only realised by those who have suffered from its absence, and it is one of the helpful objects of the association to give grants-in-aid to meet the cases where patients are unable to pay in full the fees fixed. The Home Committee of the Association engages nurses in England, and pays the entire expenses of travelling to the headquarters of the local branch, provides uniform outfit, advances three months' salary recoverable in India, and guarantees the first year's salary. The Queen is a patron, and the Duchess of Connaught is president of the association. Major-General J. Bonus is the hon. secretary.

### JUNIUS S. MORGAN BENEVOLENT FUND.

The secretary of the Royal National Pension Fund for Nurses asks us to request every nurse who has received his circular letter relating to the above fund, and who has not yet replied to it, to do so at once, especially if she does not intend to subscribe or collect. We hope to publish a complete list of the nurses who have consented to collect in honour of the Queen, and also of the subscribers to the Junius S. Morgan Benevolent Fund.

### UNSUCCESSFUL AFFILIATION.

At the recent annual meeting of the Rugby District Nursing Association, the report presented stated that "affiliation with the Queen's Jubilee Institute was found to work so unsatisfactorily that, on a resolution passed at a special meeting of the subscribers on November 5th, 1895, the association severed their connection with it. The committee were happy to say that their severance had in no way interfered with the efficiency of the work, which had been carried on by their nurse most satisfactorily." There are generally two sides to a question, and the other one in this case, as we find on referring to head-quarters, is that the "severance" spoken of was found desirable by the Rugby Association after two unsatisfactory reports had been returned to them by the Inspector of the Queen's Nurses after the yearly visit.

### FOOR LAW OFFICERS' SUPERANNUATION ACT.

A meeting is to be held at 12, Buckingham-street, Strand, on Tuesday, November 24th, at three p.m., of matrons of poor law infirmaries and others interested in the effect which the new Poor Law Officers' Superannuation Act is likely to exercise on trained nursing in workhouse infirmaries, Metropolitan Asylums Board, and all institutions which come under its provisions. It is to be hoped that the meeting will be well attended, for the matter is one of great and far-reaching importance, and the present is undoubtedly the moment to be seized upon for discussing it in all its bearings.

### LADY DUFFERIN'S FUND IN THE PUNJAB.

Efforts are being made to stimulate the work of the Punjab Branch of the Dufferin Fund, and we hear that the Government of India is taking steps to make over to it the money which is at present being expended on certain women's scholarships in the Lahore Medical School. The managing committee of the fund will be able to use this money for the assistance of students, and to devote any surplus to other ways of promoting the supply of medical relief for the women of India. Local bodies that provide scholarships for women medical students will be requested to make over the money for this purpose to the fund, and appeals are being sent out generally on behalf of its objects. One great difficulty is in finding a sufficiency of suitable candidates, and it is proposed to ask the Deputy Commissioners to give what help they can to the honorary secretary of the Punjab branch in this respect.

### SHORT ITEMS.

At the bazaar just held at Cardiff in aid of the infirmary the total receipts for the four days amounted to £3,614 3s.—The Duchess of Sutherland has sent to the Longton Sick Nursing and Samaritan Society £10, a prize won by her at a recent agricultural show.—A successful ball was lately held at the Town Hall, Sydney, in aid of the Children's Hospital at Glebe Point, Sydney.

### Hygiene: For Nurses.

By John Glaister, M.D., F.F.P.S.G., D.P.H.Camb., Professor of Forensic Medicine and Public Health, St. Mungo's College, Glasgow, &c.

### XXXIII. — INFLUENCE OF CLIMATE, &c., ON INFECTIVE AND CONTAGIOUS DISEASES.— (*Continued.*)

Diphtheria is a microbic disease which chiefly attacks the surfaces of the throat and air-passages, although it may also appear on abraded surfaces on other parts of the body. Its connection with a microbe was long suspected before the specific organism was discovered and isolated. Laycock, so far back as 1858, first made the suggestion. A committee, appointed by the Royal Medical and Chirurgical Society of London to enquire into the nature and cause of membranous croup and diphtheria, reported in 1879 that while certain micro-organisms were found in the false membrane, they did not regard them as the causative agents. Oertel, in Germany, next found a microbe of the *coccus* form, which he believed to be the prime cause. Klebs, in 1873, discovered a bacillus in the throat of affected patients, but he failed to isolate it. Friedrich Löffler next investigated the cause, found the bacillus described by Klebs, and succeeded in isolating and cultivating it outside the body. He also proved the presence of the organism described by Oertel, and established that it was not the prime factor. The bacillus of this disease, therefore, came to be called the *Klebs-Löffler* bacillus, but Koch did much to substantiate Löffler's conclusions. It principally attacks those of tender years, although those also of riper years. It attacks the female more than the male sex, and the death-rate of the former is higher than that of the latter. It prevails more in rural than in urban districts, although in the latter it would appear to be increasing, whether due to increased facilities for inter-communication or from drain causes, or both, has not yet been definitely determined. It attacks the lower animals also, notably cats, and there are reasons for believing that it is associated with certain ulcerated conditions of the udder of the cow; hence it may be propagated by milk.

The toxine which the bacilli produce during their growth in the air passages is absorbed into the circulation of the patient, and gives rise to serious symptoms, such as profound physical depression, and paralysis; indeed, this latter effect is sometimes very marked, even where the throat mischief has been but slight. Localities, and even single houses, sometimes attain unenviable notoriety as being causative of the disease.

Erysipelas, the microbe of which has been isolated as a streptococcus, is a disease which differs from the type of zymotics in that one attack does not confer immunity against a second; indeed, it would appear as if a person once attacked is rather the more predisposed to subsequent attacks. The same microbe has to do with the genesis of puerperal fever. The incidence of erysipelas is upon the skin of exposed portions of the body, and the organism gains an entrance by means of an abraded surface, sometimes very trifling in size. Popularly it is called *the rose*, from the inflamed condition of the skin. It attacks, by preference, the male sex, probably owing to the fact that males are more exposed to causes producing skin abrasions. It is most fatal in the winter months. It has practically disappeared from hospital practice before antiseptic and aseptic precautions. The infective matter is chiefly found in the affected skin.

Puerperal Fever (Lat., *puer*, a boy; *pario*, to bear) is a communicable disease associated with lying-in women. The term embraces a variety of conditions, having one feature in common, viz., septic poisoning. It is caused by the absorption of the above microbe, or of one of an allied type. Its infective character is most marked, and the infective matter clings tenaciously to the clothing and person of attendants, hence the most scrupulous care and thoroughness must characterise the disinfection of every thing and every person associated with the case. It may arise from sewer air, which carries the specific micro-organisms, hence the sanitary equipment of the lying-in room must be carefully supervised. Careful aseptic toilet of the patient, absolute cleanliness of hands and instruments, and attention to the ordinary rules of sanitation will do much to prevent its inception and check its progress.

Pneumonia, in one of its forms, must be classed among the zymotic diseases. Friedlander and Talamon, in 1882, discovered in "croupous" pneumonia a certain micro-organism, and Gamaleia has proved that this "pneumococcus," called the *streptococcus lanceolatus*, must be reckoned as the prime cause. An epidemic of this disease prevailed in Middlesborough in the first half of 1888, of which 369 persons died. Klein, who investigated the cause of this epidemic, associated it with a bacillus, called the *B. pneumoniæ*. In several particulars, epidemic pneumonia is identical with the pleuro-pneumonia of cattle.

Of other diseases of the microbic type, such as rabies, tetanus, leprosy, and malaria, much might be said. The two first are only conveyed by direct inoculation—rabies from the bite of a rabid animal, tetanus from infected earth, or other form of "dirt." They are not communicable as scarlet fever or measles is. Leprosy—due to a specific microbe—is communicable from one person to another by contagion. It is endemic in certain countries, as Norway, China, Palestine, &c. It chiefly invades the skin, in the first instance. Attempts to cultivate the bacillus out of the body have only recently succeeded. Malaria is due to micro-organisms which are located in the soils of certain districts of certain countries, which have one feature in common, viz., saturation with organic débris. It used to be endemic in the Fen districts of England, as it is to-day in the Campagna near Rome. Malarial fever has different types. Much investigation has been made as to the causes. The bacillus of Klebs and of Tommasi - Crudelli, the corpuscles of Laveran, and the amœbæ of Marchiafava, have all been considered blameworthy. The disease is not communicable from person to person. The cause usually gains entrance into the body by inhalation, and the usual vehicle is the air. It essentially belongs to the soil of the locality, but it may be conveyed from place to place in transported earth. There is no reason to doubt the possibility of its being carried from place to place by clothing or other substances which have been exposed to the infected atmosphere.

Glanders is a disease of horses. When it appears in a stud it quickly spreads, and necessitates, by law, the destruction of the affected animals, in order that the plague may be stayed. It is communicable to man by direct contagion, in whom it is ordinarily fatal, as in the case of Dr. Hoffmann, of Vienna, in 1889.

Anthrax, or Splenic Fever—so-called in the lower animals, or wool-sorter's disease or malignant pustule, its synonym in man—is usually a fatal disease of both. Thanks, however, to the researches of Pasteur, Koch, and others, a protective vaccine has been discovered which has prevented untold suffering to animals and has saved huge sums of money to agricultural economy. Its connection with man was first detected when an investigation was made into a strange disease of wool-sorters at Bradford, and in hair-sorters elsewhere—hence its name; and, again, when certain individuals who deal with the hoofs and hides of animals were attacked by an angry-looking boil, or pustule, which often proved fatal—hence the second name. Their common identity was established by the presence in each of the *bacillus anthracis* in the blood of those attacked. The spores are among the most resistant of means of destruction. All the parts of the body of the infected animal carry the bacillus; hence, blood-smeared hides, hair, or hoofs, when dried, convey the disease.

Tuberculosis is a disease common to man and many of the lower animals, and is caused by a specific bacillus, *B. tuberculosis.* To a limited degree it may be considered contagious, and to a lesser degree infectious. In cases where contagion has been proved to be the cause, two conditions have been found to prevail, viz., unhygienic apartments and propinquity of persons, as husband and wife, sisters, or brothers. It is popularly reckoned to be an hereditary disease, but it is so only in an extremely limited degree. Usually it is not directly transmitted from parent to offspring, but the predisposition is. The bacillus is found abundantly in the expectoration of phthisical persons, and in the milk of tuberculous cows when the udder is attacked by the disease; hence such dried sputa and milk may be sources of infection. A third possible source is the use of imperfectly-cooked flesh of tuberculous animals. Opinion is still much divided on this point. Some opine that the whole carcase of the animal affected even by limited tubercle is dangerous to health, others, that after extirpation of the affected organ, it may be used as food quite safely. The recent report of the Commission on Tuberculosis supports the latter view, as did also the Victorian Commission, while the Congress on Tuberculosis held in Paris sustained the former view. In consequence of this diversity of expert opinion, the action of sanitary authorities has been divided. In Berlin, where dead-meat inspection is practically perfect, a middle course is adopted. By a Ministerial decree of 1892 it was enacted that when the flesh of animals contained general tubercle the whole carcase was to be condemned; where, again, only one organ, or two organs lying contiguous in the same cavity, was affected, the carcase was not to be deemed unfit for human food, but was deemed to be flesh defective in quality. It was then thoroughly cooked, under police supervision, and sold at a reduced price. This procedure, therefore, protects the consumer, while, at the same time, it restores to the municipal economy what, under the more rigorous view, would have been lost to the food supply. This course is at once reasonable and safe from any point of view.

The propagation of tubercular disease in our infantile population must be largely attributed to tuberculosed milk. Cows confined to sheds in towns or cities are more liable to this disease than those fed in the open. Experience abundantly proves this. Hence the necessity for constant and careful supervision of cows in town cowsheds by the sanitary authorities, as is prevalent in certain cities of Denmark and other countries.

Again, from the desiccated expectoration of phthisical persons, infection is likely to arise by reason that the microbes are then capable of being air-borne, and those susceptible, of being attacked. These become important considerations when it is recollected that over the world one-seventh of the total number of annual deaths is due to the action of this microbe. Of recent years attempts have been made in various countries—limitedly, it is true—to cause disinfection of infected sputa, but many obvious difficulties emerge. In Germany, France, America, and in certain of our own cities, public attention has been called to this danger, and instructions have been printed and publicly distributed, in order to aid in minimising the danger, the main points of which are as follow: (1) That the infective material of the consumptive is located in the sputum; (2) that it is only capable of doing harm when the sputum becomes dry; (3) therefore such sputum should be received on pieces of paper, or old cotton, or linen, and burned, or received into small spittoons and mixed with sawdust before burning; and (4) when phthisis begins, the patient to be warned not to swallow the expectoration. A consumptive patient should sleep alone in a room occupied by himself solely, and the expectoration should be treated as described. We have no sympathy with the proposal to shut up within the wards of an isolation hospital everyone who is the pitiful victim of tuberculous disease, but we advocate strongly the disinfection of the personal and bed clothing, and room, of every person whose death has been registered as due to tubercular phthisis. Such measures, coupled with an increase of open spaces in our cities, closer attention to ventilation of occupied rooms, greater insistance on smoke consumption, and rigorous periodic examination of the milch cows of town sheds, would undoubtedly assist in lowering the spread of this baneful malady.

### Our American Letter.

The jubilee of anæsthesia was fitly celebrated on October 16th at the Massachusetts General Hospital, in Boston, where, fifty years ago, the first operation under ether was performed by Dr. John C. Warren; Dr. William T. G. Morton, the young dentist whose discovery of ether as an anæsthetic has been such an inestimable blessing to mankind, being the administrator. It was curious that the invitations should have been sent out by another Dr. Warren, a grandson of the surgeon who helped to make Dr. Morton's great discovery generally known, and that these invitations should be signed on behalf of the trustees of the hospital by Dr. W. S. Bigelow, grandson of another surgeon of the hospital who was present at that famous first operation. Amongst other distinguished guests Lord and Lady Playfair were present. An interesting description of the first experiment was given by Dr. Davis.

In October the new medical hospital building of the New York Homœopathic Medical College was dedicated and opened. The new building has cost some 100,000 dols., and has been erected in order to furnish special clinical opportunities to the students. Many new hospitals and institutions are springing up in various parts of the States. Among them may be mentioned the Alexian Brothers' Hospital, of which the corner stone was laid at Chicago last month. The building will be replete with every modern requirement, and devised after the most approved methods, with rounded corners, and innocent of all dust-traps. It is expected that the cost will be about 250,000 dols. Amherst College, Brooklyn, has been the fortunate recipient of a generous gift from Mr. George D. Pratt, in the shape of a proposed new hospital, with special isolation wards, to be erected about half a mile from the college. A new hospital for contagious diseases is almost ready for occupation at Worcester, Mass. It will provide two pavilions—one for scarlet fever and one for diphtheria patients—with a two-storey administration building, a special building for a steam disinfector, and a well-equipped laundry.

The Malden Hospital, Mass., is regretting the loss of its Superintendent, Miss Bliss, who has resigned her post after four years' good work, during which time the training school has increased in numbers and in efficiency. The trustees presented Miss Bliss with a most cordial testimonial on her departure. Miss Whitmore, of Salem, succeeds Miss Bliss.

The nurses of the General Hospital, Buffalo, New York, have been heartily glad to welcome back, after a two months' absence, their superintendent, Miss Kennedy. It was feared that Miss Kennedy's health would compel her to give up her work, but she has now been able to return to her post.

### Nurses in 1896—Their Quarters, Hours, and Food.

[These articles exhibit the actual condition of affairs in the spring of the present year.]

### POPLAR HOSPITAL FOR ACCIDENTS.

### I.—Terms of Training.

Probationers are received at the Poplar Hospital for Accidents, where excellent training in surgical and accident work is to be obtained, for a term of two years' training. If appointed after a month's trial they are required to sign an agreement for two years' service, and this they cannot break except by the special permission of the committee. Should the committee, on the recommendation of the matron, wish to terminate the engagement they can do so upon payment of salary due and three months' salary in advance; in case of dismissal for fault only the salary due will be paid. Regular probationers must be between the ages of twenty-two and thirty. They are required to take day and night duty alternately, the average proportion being three months on night duty to five months on day duty.

A few paying probationers are received for periods of three months on payment of £13 13s. in advance. These probationers are not required to go on night duty unless they wish to do so.

Courses of lectures are given by the matron and house surgeon, which probationers are required to attend, and for which time is allowed from the wards.

### II.—Hours of Work and Times off Duty.

The interest taken in hospital and nursing matters by the chairman of the Poplar Hospital, the Hon. Sydney Holland, is matter of public knowledge, and at the hospital with which he is most intimately connected it may be well imagined that the comfort and well-being of the nursing staff are considered in every way. The approximate number of hours in the wards on day duty for the nurses is ten, counting, that is, the minimum off duty time of two hours, besides meal times. Nurses go on duty in the morning at half-past seven, leaving their wards at nine o'clock. During these hours half an hour is allowed for dressing after the morning work, time for lunch, and half an hour each for dinner and tea. Two hours off duty is the least amount of time allowed for recreation each day; very frequently three hours are given, as the work allows. Besides this, probationers have three half-days, and one whole day—a real whole day, that is, not one with all the good taken out of it by two or three hours' hard work in the early morning, each month. The half-days (eight hours) are taken alternately, in the morning and afternoon, and in the former case breakfast in bed is allowed. On the day preceding a day off probationers may take their two hours from seven to nine in the evening, and sleep away from the hospital if they wish. Staff nurses and sisters have a half-day each week, and a whole day (a Sunday) once a month, the whole day following the half-day so as to allow nurses to go right away. By permission a staff nurse or sister can occasionally take her monthly day off in conjunction with the half-days of the preceding and the following week, so that leaving the hospital on Saturday afternoon she need not return till midday on the Monday, thus securing two nights away from the hospital, a holiday of sufficient length to be a real refreshment and rest. At Poplar—a custom which would seem to be unique—nurses and sisters are given 5s. on the occasions when they take a whole and a half day together, to help towards the expenses of going away, so important are these holidays felt to be in keeping the staff in good health. Miss Bland, who is a very ready co-operator with Mr. Holland in giving her nurses as much liberty as possible, says that the improvement in the health of the staff since it has been found possible to give these liberal times off duty has been most marked, and it is now very much the exception for any nurse to knock up. The hospital and the patients, no less than the nurses, are therefore gainers. The sisters' daily hours off duty are usually from five to seven, but these naturally vary with the work, and depend upon the hours of the visiting staff, which at a small hospital like Poplar are not as regular as at the large general hospitals. Night nurses are on duty from nine p.m. to nine a.m., having two and a-half to three clear hours for recreation and rest every day, besides meal times, in the morning or evening, as they prefer, and one night a month. "Theatre leave" is given at the Matron's discretion. Every member of the staff is allowed a month's holiday in the year.

### III.—Meals.

Sisters and day nurses breakfast at seven a.m., there is a light luncheon in the ward kitchen between nine and ten, dinner is at half-past one (the sisters lunching at ten minutes to one, and dining with the matron at seven p.m.). Tea is taken in the ward kitchen, the sisters having theirs in the dining-room, and the nurses' supper is at nine p.m. The matron presides, and lunches at the nurses' mid-day dinner. Night nurses breakfast at half-past eight p.m., and dine at half-past nine a.m. The food for the nurses' night meal is sent each night to the ward kitchen, and till wanted can be kept in a larder cupboard outside the window or in the ice-box outside the ward. A small cupboard is devoted to the nurses' tea-things, and a weekly cake is sent to each ward for the nurses. Meals at Poplar are well cooked and nicely served, appointments including table napkins. Eggs and butter are specially procured from the country. The dietary book shows that the food is varied as much as possible. Breakfast is a meat meal, with jam or marmalade; hot meat, with puddings or tarts, is provided for dinners; and supper consists of soup, hash, maccaroni, cheese, &c., with tea or milk. Ale, stout, milk, and lemonade is provided for dinner.

### IV.—Salaries and Uniform.

Probationers are paid £12 the first year and £22 the second. When appointed staff nurse the salary received begins at £25 a year, rising £1 annually to £30. Sisters' pay is from £30 to £35; that of the night sisters £35 to £40.

Indoor uniform is provided by the hospital, two galatea dresses a year for the nurses, dark blue drillette for the sisters, with twelve yards of linen for aprons, and three caps. None of the staff are required to wear outdoor uniform. The expense of the nurses' washing is borne by the hospital.

### V.—Nurses' Quarters.

The quarters allotted to the nurses are in the main building and are very comfortable. Every sister, nurse, and probationer has a separate room, furnished with a liberal regard to comfort. Sisters have no sitting-rooms near their wards, and their rooms are therefore arranged to serve as sitting as well as bed rooms, and are provided with an easy chair or sofa and screen, being cosy little apartments. Sitting-room space is at present somewhat cramped, a difficulty which will be removed when the new block now being added to the hospital is completed. More bed-rooms and another sitting-room will then be available. There is now only one dining and sitting room, but that is pretty and home-like, well lighted, and airy, on the top floor not far from the kitchen. There are three baths, and more will be added when the new building is completed. Altogether the nursing staff at the Poplar Hospital for Accidents are to be very much congratulated upon the care for their comfort and happiness which is evident in every detail of the arrangements, and which has been certainly due to the great personal attention and keen interest given to the matter by Mr. Holland.

### Midwifery Papers.

### IX.—HÆMORRHAGE: ANTE-PARTUM.

Ante-partum Hæmorrhage, or hæmorrhage occurring before delivery takes place, is the most serious complication of pregnancy. It is caused by the partial separation of the placenta from that portion of the uterus to which it is attached. In the early months of pregnancy hæmorrhage is a frequent cause of abortion, and when it occurs in the later months is a serious danger to both mother and child. There are two kinds of ante-partum hæmorrhage: (1) unavoidable or placenta-prævia; (2) accidental.

(1) Unavoidable hæmorrhage is caused by the placenta becoming attached to the lower part of the uterus near the os, instead of its usual site, the fundus. This condition is called placenta-prævia, and generally occurs in women who have borne several children from enlargement of the uterine cavity, or some morbid condition of the uterus. The three varieties of placenta-prævia are: (1) placenta situated entirely over the os; (2) partly over os; (3) over the lower segment of the uterus near the margin of the os. In placenta-prævia the hæmorrhage occurs suddenly, without a previous history of accident or injury. It may come on at night during sleep. The loss varies considerably; at first the hæmorrhage may be quite slight, and may stop of itself, but it generally recurs suddenly in a few days, or perhaps weeks' time, and each successive loss is more profuse. The seventh month is the most usual time for this hæmorrhage, but it sometimes occurs earlier in the pregnancy and causes abortion. Placenta-prævia is generally suspected when hæmorrhage occurs without any appreciable cause. Examine vaginally, and you will find the os slightly open with the placenta or placental margin presenting. Sometimes a large clot of blood retained inside the os may be mistaken for the placenta, but this clot will be found easily breakable under the pressure of the finger, unlike the placenta, which is much firmer. Treatment: Send for a medical man at once. If the loss is not great keep the patient in bed, give cold drinks or ice to suck, keep the room quiet and airy, and do not allow the bed-clothes to be too heavy. On no account leave your patient till the doctor arrives, for at any moment an increased flow may set in and her life may be in jeopardy. If severe hæmorrhage occurs while you are waiting, you must act at once. In all cases when the loss is great rupture the membranes as soon as they can be reached. Get your finger past the placenta and try to reach the presenting part of child. If the os is not sufficiently dilated to admit of this, plug the vagina close up to it to excite uterine contractions. If you can succeed in rupturing the membranes plug the vagina immediately afterwards to still further stimulate uterine contractions and dilatation of the os. Your object is to induce labour and by the contraction of the uterus to close the bleeding blood vessels. Strips of surgical lint or packed cotton-wool or a clean white silk handkerchief may be used as plug. Do not allow the plug to remain in the vagina longer than a few hours. Put on a firm binder and try to excite uterine contraction by occasional friction. Take note of the pulse and general condition of the patient. On no account give stimulants unless there are marked symptoms of collapse.

(2) Accidental hæmorrhage is the most serious form of ante-partum hæmorrhage. It is caused by the partial separation of a normally situated placenta from the maternal site through over exertion on the part of the mother, lifting, straining, or a fall or knock. A diseased placenta is occasionally the cause of separation. There are two kinds of accidental hæmorrhage —(1) revealed and (2) concealed. In the first the blood escapes through the os-uteri; in concealed hæmorrhage, which is the most dangerous form, the blood is retained in the uterus and the amount of visible loss is not great. This retained hæmorrhage renders the condition more serious by distending the uterus still further and preventing its contraction and the constriction of the blood-vessels. The patient complains of "dragging and tearing" pain, but the feeble and quick pulse, great restlessness, anxiety of expression, sighing respiration, blanching and coldness of face and extremities, and general signs of collapse are more prominent symptoms and mark great loss of blood. Treatment: If the loss is not great keep the patient quiet till the medical man arrives, but convince yourself that the slight flow from the os is not a sign of concealed hæmorrhage. If the loss is great and the hæmorrhage is concealed, puncture the membrane as soon as possible and remove any large clots you may feel inside the os. Do not plug the vagina, but excite uterine contraction externally by pressure and friction, and apply a tight binder. If the collapse is marked give a stimulant, keep the patient's head low, and place a hot bottle to the feet if cold. Keep a firm pressure on the fundus the whole time.

### Cookery Classes at the London Hospital.

For the past year the London Hospital probationers have enjoyed the advantage of a resident teacher of cookery, who is a first-class diplomée of the Edinburgh Training School of Cookery. A knowledge of invalid cookery now forms part of the training of every probationer, and there are always thirty pupils in training, twenty from the Preliminary Training Home, Tredegar House, and ten nurses or probationers from the hospital, who began their nursing career before the establishment of Tredegar House. The course is six weeks, and the syllabus, drawn up by the National Training School of Cookery, consists of twelve lessons. Two demonstrations are held every week, which are attended by all the students, each of whom must also attend two practice classes of two hours each in the week. In the seventh week, at the conclusion of the course, an examination is held. The dainty dishes cooked in the practice classes become the property of their makers, and are much enjoyed for supper, or by the patients in the wards, to whom they often find their way. The syllabus is as follows:—

### Courses of Lectures on Sick Room Cookery.

I.—Vanilla Soufflée; Beef Tea; Custard Pudding; Fried Sweetbread.

II.—Light Tea Cakes; Boiled Chicken and Egg Sauce; Fried Filletted Plaice; Sweet Omelet.

III.—Chicken Broth; Grilled Chop and Ribbon Potatoes; Stewed Eels; Tapioca Pudding.

IV.—Port Wine Jelly; Fried Sole; Scones; Scrambled Eggs; Fried Bacon.

V.—Shin of Beef Soup; Sole à la Maitre d'Hôtel; Milk Jelly; Poached Eggs; Toast Water.

VI.—Roast Chicken and Bread Sauce; Florador Pudding; Stewed Oysters; Lemon Sponge.

VII.—Lemon Jelly; Arrowroot Pudding; Fish Cakes; Quenelles.

VIII.—Restorative Soup; Boiled Sole and Sauce; Empress Cakes; Lemonade; Wine Whey.

IX.—Stewed Ox Tails; Apple Jelly; Boiled Custard; Œufs à Madame.

X.—Oyster Soufflée; Cornflour Blanc Mange; Bread and Butter Pudding; Savoury Omelet.

XI.—Sponge Cake; Rissoles; Coffee; Stewed Tripe; Savoury Custard.

XII.—Rice Moulds; Sole au Gratin; Pancakes; Gruel; Porridge; Barley Water.

### Council of County Nursing Associations.

A conference, under the auspices of the Council of County Nursing Associations, was held, by permission of the Duchess of Sutherland, at Stafford House on the 17th inst., the Earl of Winchilsea presiding. The conference was so largely attended that it was found necessary to hold the meeting in the Staircase Hall, the room in which the members first assembled being too small to accommodate them. Among those present were the Duchess of Sutherland, the Countess of Winchilsea, the Countess of Selborne, Lady Baker, Lady Belper, Lady Buxton, Lady Barttelot, Lady George Campbell, Lady Falkland, Lady Mary Holland, Lady Le Marchant, Lady Parker, Lady Priestley, Lady Rayleigh, Lady Laura Ridding, Lady M. Wilson, the Hon. Mrs. H. Currie, Hon. Mrs. C. Egerton, Hon. Mrs. Howard, Hon. Mrs. Randall, Hon. Mrs. Freeman Thomas, Miss Broadwood (founder of the Ockley system, representing forty-three nursing institutions working on that system), Miss Wintmore Jones (representing two), Sister Katherine (Plaistow), Lord Valentia (Chairman of the Oxfordshire County Council), Hon. Sydney Holland, Dr. Thresh, and various representatives of County Councils.

The Chairman congratulated the meeting on the fact that representatives from about sixteen County Councils and a very great number of nursing associations in all parts of the country had attended. Tracing the history of rural nursing up to the present day, the Chairman said there had been two systems at work—the "Ockley" system, under which village women, after a very short training, were sent to nurse one case at a time, living with the patients and doing the housework; and the district nursing system of the Queen Victoria's Jubilee Institute for Nurses, under which a fully-trained nurse was placed in charge of a district, and undertook a number of cases at the same time. The former system (which owed its institution to the indefatigable energy, zeal, and courage of Miss Broadwood, and which had done the best which was possible under the circumstances, with very little assistance), as a complete and satisfactory system for the whole nursing of a rural district left much to be desired. For instance, no sufficient minimum standard of training had been adopted, no recognised periodical inspection of the nurses was made, and inasmuch as the system was confined to cottage nurses, and did not employ fully-trained nurses, no attempt was made to deal with the problem of the nursing of the large number of small towns which were to be found in rural districts. The other system was too expensive for a rural district, for something less costly than a highly trained Queen's nurse must be placed at the disposal of the poorer districts, or else such districts would have to be excluded from any nursing scheme. Two things had happened recently which had helped to bring the two systems nearer to one another. The first was the granting of nursing scholarships by County Councils, which to a certain extent obviated one great difficulty of the past—the want of funds for training the nurses. The second was the decision of the Queen Victoria's Jubilee Nursing Institute to recognise and inspect nurses who had received a minimum training for six months in district work and midwifery and who held the L.O.S. certificate. His conclusions on the subject were that great public benefits and great help to rural nursing would follow the granting of County Council scholarships for the preliminary training of nurses under proper restrictions; and that in order to justify these grants there must be a responsible association to employ the nurses, a minimum of training, and a periodical inspection by some competent body. He mentioned that in his own county—Lincolnshire—all the points to be aimed at had been carried out. In conclusion, he trusted that the outcome of the present conference would be to carry them a step further towards building up a system which with the greatest economy and the greatest efficiency would bring trained nursing within the reach of the sick and suffering poor.

Mr. Sutton Nelthorpe (chairman of the Technical Education Committee of the Lindsey County Council) opened a discussion on the granting of scholarships by County Councils for the preliminary training of nurses to be employed by county nursing associations. His County Council had for the last three years given four scholarships of £35 a-year each to nurses who should be selected through the agency of the County Nursing Association, and an additional £100 a year for the further training of these nurses in general nursing of the sick. One point only of the original scheme, viz., that the nurses selected should undertake to serve for three years in the county, had been objected to on the ground that Clause 8 in the Technical Education Act of 1889 forbade the teaching of any trade or employment by County Councils, and so the condition of service with the County Council had been eliminated, the Council leaving any conditions of that kind to be made by the association. No vote passed by his County Council had been more popular than that providing the funds for these scholarships, and if any objections were raised to their spending money for that purpose great good would be done, as attention would then be drawn to Clause 8 of the Technical Education Act of 1889, under which, although considerable sums were placed at the disposal of the local authorities for the purposes of technical education, the Councils were practically forbidden to use them for any useful purpose.

Lord Valentia said that his County Council had intended to help the scheme of rural nursing as much as possible by the granting of scholarships, but the attempt was nipped in the bud by the way in which Clause 8 was read by the Science and Art Department.

Alderman Snape (chairman of the Technical Education Committee of the Lancashire County Council) did not see how the difficulty of Clause 8 was to be overcome. His County Council was quite in sympathy with the movement.

Lady Baker (hon. secretary of the Dorset Nursing Association) gave a brief account of how nursing scholarships had been granted by the Dorsetshire County Council, and the conditions under which they were granted.

Mr. Harvey (chairman of the Technical Committee of the Suffolk County Council) doubted the legality of nursing scholarships, but advised all County Councils to go on making grants in aid, for he felt sure that no auditor would surcharge the county on account of them.

Mr. Wiltshire (organising secretary of the Herefordshire Technical Education Committee) said his County Council had decided that as the nurses would be going to get their living by the training they received, it would not be legal to found the scholarships.

The Chairman said that even were the items disallowed by the auditor, he had been given to understand that on the first occasion of the surcharge being made, if the Board of Trade were satisfied that the expenditure had been incurred on public grounds, they would remit it.

The next subjects for discussion was the minimum training requisite, and the question of inspection.

The Duchess of Sutherland detailed the system which had been pursued under her auspices in Sutherland, where the district nurses had been trained under the "Ockley" system, with variations suited to the peculiar needs of the district. The minimum training necessary, in her opinion, was six months, and the nurse should hold the L.O.S. diploma.

The Hon. Sydney Holland said that the Q.V.J.N.I. fully recognised the different conditions of things in the town and in the country. They felt that in both cases the nursing must be good, and they were therefore willing to inspect the work of nurses with a minimum training of six months, with the proviso, however, that each class of nurse should within reasonable limits be confined to that class of work for which it was intended.

Dr. Lowe said that, although their nurses only received initially six months' training, yet, as they were under the inspection of the Q.V.J.N.I., and of the local medical men, at the end of two or three years they were nearly, if not quite, as fully trained as if they had been all that time in an institution.

Miss Broadwood, the founder of the Ockley system, traced the history of that system from its foundation fourteen years ago.

Lady Laura Ridding said that the villages had a strong claim on the Q.V.J.N.I. The money the Queen gave to that institute consisted of the pence from the villages as much as of the pence from the towns.

The Chairman suggested that as the meeting had lasted so long already the other subjects on the agenda, viz. : "The establishment (*a*) of a centre in London where information on rural nursing throughout Great Britain may be obtained and (*b*) a registry of county nurses in connection with it," and "The best means of securing, as far as possible, uniformity in the working of county nursing associations throughout the country," had better be remitted to the council so that they might take such steps as might be expedient in the matter.

This having been agreed to, a vote of thanks brought the meeting to a close.

### Appointments.

### MATRONS.

Dawlish Infirmary.—Miss Fittall has been appointed Matron at the Infirmary, Dawlish. She was trained at Manchester Royal Infirmary, and has since worked at the Retford and Ulverston Cottage Hospitals.

Doncaster Infirmary and Dispensary.—Sister Florence Longrigg, of Mildmay, has been appointed Lady Superintendent of the Doncaster Infirmary in succession to Sister Josephine Gelston, who has retired after many years' service. Sister Florence was trained at Crumpsall Infirmary, and has since worked as ward sister at Mildmay Memorial Hospital, as private ward sister at Friedenheim, and as superintendent of the Blandford Cottage Hospital.

Windsor and Eton Royal Dispensary and Infirmary.—Miss Helen E. Court has been appointed Matron of this hospital. She received her training at Guy's Hospital, and at the Hospital for Women, Soho Square, London; she was sister at Charing Cross Hospital, and afterwards sister at the Leicester General Infirmary for four years, the greater part of the time in charge of the large accident ward (42 beds). Miss Court has since held the post of matron for two and a half years to the London Central Throat and Ear Hospital, Gray's Inn Road.

### Minor Appointments.

Birmingham and Midland Eye Hospital.—Miss Ethel Mathews was appointed Charge Nurse of the children's ward at this hospital on November 16th. Miss Mathews was trained at the Warnford Hospital, Leamington.

Metropolitan Convalescent Institution, Broadstairs (Children's Branch).—Miss E. M. McNamee has been appointed Sister at this institution. Miss McNamee was trained at the Hospital for Sick Children, Great Ormond Street, where she subsequently held the post of staff nurse in Helena Ward.

### The Windsor and Eton Royal Dispensary and Infirmary.

### A MATRON WITHOUT ADEQUATE AUTHORITY.

For some time affairs have not been working very smoothly at the Windsor Infirmary, and ultimately resulted in the resignation of Miss Coke, the matron. The appointment of her successor has been attended with some difficulty, as the arbitrary wording of a rule relating to the appointment and dismissal of nurses and servants has caused friction. This rule, No. 17, as it at present stands, states: "That the committee are empowered to appoint honorary visiting surgeons to manage the ordinary details of the institution, and to appoint, suspend, or dismiss any paid official or servant of the institution." As the selected candidate, Miss Bolton, who was regarded as in every way suitable to fill the office of matron, withdrew her application, considering that Rule 17 interfered with her control over her department, the Chairman of the Infirmary (the Rev. Canon Ellison) suggested that it should read, "This rule shall not apply to the appointment and dismissal of the nurses, probationers, and servants, which shall be left in the hands of the matron." A meeting of the Governors was accordingly summoned for the 13th instant, to consider the resolution, Mr. F. T. Barry, M.P., taking the chair. A notice of the meeting and of the resolution was published in the Windsor papers, but before the meeting Canon Ellison made inquiries as to the custom which obtained at other well-regulated institutions of the kind, his inquiries going to show that the management of the nurses and of the domestic staff is usually vested in the hands of the matron, but at the same time the confirmation of the appointment and dismissal of nurses and servants is left to the committee. A Governor enquired, at the opening of the meeting, if an alteration in the resolution might be suggested, but all amendments were ruled out of order by the chairman. The resolution before the meeting, therefore, placed the entire control of domestic matters in the hands of the matron. The suggested alteration of the rule and the limitation of the authority of the committee brought down a good deal of indignation upon Canon Ellison's head at the meeting, a member of the weekly board going so far as to say that he regarded the resolution as a vote of censure upon them, and that should it be passed by the meeting they would resign. A governor pointed out that as the matter referred to the committee only, and not to the weekly board, there appeared to be little reason for the latter's attitude in the matter. Ultimately Canon Ellison withdrew his resolution, so that it is difficult to understand how a wise and competent matron can accept office, and the administration of this institution must suffer unless the rule be altered. Another difficulty arose out of the proceedings. Miss Bolton was the approved candidate at the meeting held to elect the new matron, Miss Court being second on the list. Miss Bolton, however, withdrew from her candidature on account of Rule 17, but Miss Court consented, we are sorry to hear, to work under it. Canon Ellison, feeling that Miss Bolton was the more suitable candidate, wrote to her again, explaining to her that much more authority was given to the matron than the rule implied, and asking her if, under these circumstances, she would reconsider her decision. Miss Bolton then consented to do so. A special general meeting of the committee to decide between the two candidates was held on Tuesday, the 17th inst., with the result that Miss Court was elected.

This institution is the local hospital of the Queen and H.R.H. Princess Christian. It is certainly unfortunate in these circumstances that the sixtieth year of the Queen's reign should find the Windsor Hospital so out-of-date and ill-regulated. This is a case where H.R.H. Princess Christian might, out of fulness of knowledge, enlighten the managers and secure the necessary reforms.

### Everybody's Opinion.

### INFORMATION WANTED.

"Nurse C. J." writes: May I suggest it would be most useful to private and district nurses if papers were published in your "Nursing Mirror," written by "sisters" in hospitals, on the newest and best ways of carrying out various nursing details, such as sponging patients for reduction of temperature, &c.; nutritious and other enemas, their mode of preparation, &c.; the diet and special requirements of various diseases; new dressings, their properties and uses, &c. When a nurse is cut off from hospital life she is apt to become rusty, and lose by not having opportunities of hearing and seeing these things.

[We publish the above letter in case any of our contributors or others may like to follow the suggestion of "Nurse C. J." For our own part, we think the field is covered by existing publications, and anything new is noted in the Nursing Supplement from time to time.—Ed. *T. H.*]

### THE DANGERS OF INFECTION.

"Interested" writes: May I ask you to tell me whether it is a perfectly legitimate thing for the matron of a nursing home to supply a hospital with night nurses for infectious cases and to allow those nurses to return to the home daily to sleep and still wearing the same clothes and to mix with the other inmates of the house; and also, still wearing the same dress, to go to other cases? This, I am given to understand, occurred recently in one of our fashionable south-east watering-places, supposed to be fortunate enough to possess "the best M.O.H. in England." I should like to know if this sort of thing is usually done, and if the authorities usually wink at it. It seems to me to be an abominable shame, and I am astonished that such a thing should be allowed. Surely, were this made known, such homes would be boycotted, since, of course, people do not generally care to have anyone straight from infection in their houses. Who is supposed to protect the public, and who is to blame in this matter—the M.O.H., the hospital authorities, or the matron of the home?

*** The M.O.H. in question writes: The hospital referred to is a general hospital, which occasionally admits diphtheria cases, especially those requiring tracheotomy. The statements made as regards the nursing of these patients are most misleading and inaccurate. Every care is taken to prevent any danger of infection.

### ORPHANS CAGED.

The Rev. Frank T. Mills, The Rectory, Broadstairs, writes: My attention has been drawn to certain correspondence in your paper *re* the Kilburn Sisters. I should not myself have taken any notice of an unprovoked attack by one who is either afraid or ashamed to sign his name had not the "clergy of Broadstairs" been more or less appealed to, as I feel quite confident that no honourable man, and certainly no member of such a noble profession amongst which, I presume, your paper circulates, would pay any heed to an anonymous correspondent. But for the above reason I feel called upon now, as rector of this parish, to give my evidence as to the case before your readers. In the first place, I feel compelled to point out, after your very courteous footnote in the issue of October 7th—viz., "The defenders of the Sisters of the Church . . . have evidently but one scribe amongst them if we may judge by the wording of the letters which reach us"—that I do not know who "Nurse A. B." is, and that I have not consulted Dr. Raven concerning this letter. As to the drawing of the "cages," as your correspondent calls them, I am glad to say that during the time I have known the Sisters of the Church and their work—which is no short time, I have never seen a "cage" like that depicted in your paper. On seeing the drawing, I immediately went up to St. Mary's Orphanage and again examined the cubicles to be quite accurate. The cubicles are not enclosed at the top; and even suppose for one moment, for the sake of argument, that the Sisters left their charges at the alarm of fire, it would be quite possible for the children to climb out—not only possible, but I believe I am right in saying that the girls have been known to climb out of them. Then, again, they are not confined as to space. There is room for bed and other toilet necessaries. The cubicles are decorated with texts, photographs, and other ornaments not usually found in cages of a travelling menagerie. I am quite sure if your readers could only see the pains and delight these children take in decorating their cubicles, they would agree that to call them "cages resembling those of a travelling menagerie" is a cruel and groundless slander, which, as Dr. Raven very truly points out, picture to the public mind cages filthy and free from comfort. These cubicles are certainly not filthy, and the children's pride in them surely is a sufficient proof that they are not without comfort. As to the question of fire, it has been proved by practice that the children can be removed without a stampede (which would necessitate the crushing of the little ones probably to death) in the least possible time. In conclusion, I may say that a brighter, happier, healthier set of children could not be found than the inmates of St. Mary's Orphanage. This could not be if the children were "cribbed, cabined, and confined in cages, &c." I come across the children very often, and no outsider can have more knowledge of them than Dr. Raven and myself.

*** It seems a pity that our correspondents will not stick to the point. If the statements made by a "Medical Correspondent" are correct there is an end of the matter, and the whole system is condemned. If they are incorrect, why not point out the error? He said that the infants who sleep at the St. Mary's Home, Broadstairs, "are 'cribbed, cabined, and confined,' each within its own enclosure of iron bars, from which no escape is possible till the clips which fasten the prison doors are withdrawn." He also gave further particulars, and stated that these cubicles bore "a strong resemblance to the cages of a travelling menagerie." The latter simile seems to be the cause of all the anger and indignation, but we should have thought that the statement that these infants were locked up at night in wire cages was a far more serious accusation. It was in the hope of receiving some official denial of this, or some promise that it should be altered, that we opened our columns, and not for the sake of publishing praises of the sisterhood and denials of things that had never been charged against them. A "Medical Correspondent" never said the cages were filthy; he never said they were closed at the top; he never said they were deficient in space, or in air; he never said they contained no bed; he never said there was not room for toilet necessaries. All these things have nothing to do with the accusation, and are mere imaginings set up to be knocked down again, and tend to withdraw attention from the real cause of complaint, viz., that the orphans are locked up in these cages all the night. The only reasonable excuse—one, however, that is quite untenable—that we have so far seen suggested for the existence of these cages is that they would prevent "stampede" in case of fire. This is repeated by Mr. Mills, and yet in the same letter he gravely informs us that the children can climb out of the cages! By that admission the very last excuse for the existence of the cages is knocked away.—Ed. *T. H.*

### Where to Go.

Toynbee Hall, E.—On Tuesday, November 24th, Dr. Stephen Mackenzie will give a lecture to members of the Toynbee Nursing Guild on "What Doctors Expect from Nurses." Sir V. K. Barrington will take the chair at eight p.m.

Trained Nurses' Club.—The annual sale of work on behalf of the Trained Nurses' Club will be held this year, by kind permission of Mrs. Lorent Grant, at 19, Nevern Square, close to Earl's Court Station, from two to seven o'clock, on December 2nd. The "Club" stall will be presided over by Mrs. Nichol, the secretary, and "Nursing Notes" stall by Miss Brierly. Articles left unsold will be on view at Nevern Square from ten to one o'clock the following morning, and at 12, Buckingham Street, on Friday, December 4th, from half-past two to ten o'clock. On the 2nd entertainments will be given during the afternoon, under the direction of Miss Ethel Robinson. Mr. R. U. Anders, Miss Lucy Broadwood, Miss Branston, Miss Grainger Kerr, Miss Gethen, and others have promised their assistance.

### For Reading to the Sick.

### THE CONSECRATION OF SUFFERING.

**Verses.**

Lord, a whole long day of pain now, at last, is o'er!
Ah! how much we can sustain, I have felt once more!
Felt how frail are all our powers, and how weak our trust;
If Thou help not, these dark hours crush us to the dust.
Could I face the coming night if Thou wert not near?
Nay, without Thy love and might I must sink with fear;
Round me falls the evening gloom, sights and sounds all cease,
But within this narrow room, night will bring no peace!
—*Keble.*

God sends sometimes a stillness in our life—
The bivouac, the sleep—
When on the silent battlefield, the strife
Is hushed in slumber deep;
When wearied hearts exhausted, sink to rest,
Remembering nor the struggle, nor the quest.
He giveth rest, more perfect, pure, and true,
While we His burden bear;
It springeth not from parted pain, but through
The accepted blessing there;
The lesson pondered o'er with thoughtful eyes,
The faith that sees in all a meaning wise.
—*L. Fletcher.*

All ye who seek a comfort sure
In trouble and distress,
Whatever sorrow vex the mind,
Or guilt the soul oppress.

Jesus, Who gave Himself for you
Upon the Cross to die,
Opens to you His sacred Heart,
Oh! to that Heart draw nigh.
—*Latin Hymn.*

**Reading.**

Underneath are the Everlasting Arms!

My son, suffer me to do with thee what I please; I know what is expedient for thee.

I will show him how great things he must suffer for My name's sake.—*Acts* ix. 16.

Lord, behold, he whom Thou lovest is sick.—*John* xi. 3.

You, who used to be such a successful worker, are now laid by, and you are fretting for your work. Work means a sphere in which love of power, vanity, gadding about, independence, excitement, and self-love find full scope in the path of duty. No one who appreciates these delights can wonder at your feeling it a hardship to be transferred to a post where there is only scope for patience, self-control, courage, and the love of God. Of course, the workshop, equally with the sick room, affords scope for saintliness, but neither work nor sickness are saintly in themselves; that depends, in both cases, on the will being conformed to that of God. You were working for God in old days, exactly in proportion as you are now willing to resign that work at His call. In proportion as you now fret for it, you were working for your own pleasure. I do not say that it is wrong to regret that or any other lawful pleasure, but is it not the case that you, who are too religious to repine at losing gaiety, feel it allowable (and almost commendable) to repine at losing work, which is often really only a more subtle form of dissipation? Your own rebelliousness proves that I am not doing your work an injustice. If you were really doing it to please God, and He tells you that inactivity pleases Him better, you will only feel with Ignatius Loyola, "Hoc vult Deus," and be at peace. Judge the real value of your past work by the spirit in which you take your present inactivity.

### Nursing in L.C.C. Asylums.

"London," a weekly illustrated paper, is championing the cause of "Overworked Nurses" in the asylums under the London County Council, where the hours for all attendants are stated to be impossibly long, and publishes in a recent number an interview with the Hon. Sydney Holland regarding the hours of hospital nurses. Mr. Holland's views on the subject are practically interpreted at the Poplar Hospital, with which institution our article on "Nurses in 1896" deals this week. A working day of from nine to ten hours is found practicable and satisfactory from every point of view at Poplar, at King's College, at St. Thomas's, and at other voluntary hospitals. At the rate-supported asylums of London the hours of the unfortunate attendants average fourteen daily, with one day off a week. And these hours the London County Council have just refused to shorten.

### Death in Our Ranks.

The death of Sister Meheux, of the Gibraltar Military Station Hospital, from pneumonia, contracted whilst nursing a soldier patient, has caused much regret at Gibraltar, where she was known and loved. The funeral took place on Tuesday, November 10th, with full military honours. The coffin, covered with the Union Jack and beautiful flowers, was drawn by four mules on a gun carriage, Surgeon-Colonel Catherwood and other military doctors acting as pall-bearers.

### Notes and Queries.

**The contents of the Editor's Letter-box have now reached such unwieldy proportions that it has become necessary to establish a hard and fast rule regarding Answers to Correspondents. In future, all questions requiring replies will continue to be answered in this column without any fee. If an answer is required by letter, a fee of half-a-crown must be enclosed with the note containing the enquiry. We are always pleased to help our numerous correspondents to the fullest extent, and we can trust them to sympathise in the overwhelming amount of writing which makes the new rules a necessity. Every communication must be accompanied by the writer's name and address, otherwise it will receive no attention.**

*Dispensing.*

(40) Can you tell me for what examinations in dispensing women are eligible and where the papers concerning such examinations can be obtained?—*Enquirer.*

See reply to similar query in this column last week. Write to the Secretary, Apothecaries' Hall, Blackfriars, E.C., or to the Secretary of the Pharmaceutical Society of Great Britain, Bloomsbury Square, W.C., for information regarding examinations.

*The Nurse Dolls.*

(41) We are about to have a sale of work connected with a hospital. Are the "Nurse Dolls" let out on hire now for such occasions?—*Pansy.*

We are sorry that the Nurse Dolls are not at liberty just now to undertake a visit to the country.

*Male Nurse.*

(42) "William" is reminded of our rule that anonymous queries cannot receive attention in this column.

*Books for Nurses.*

(43) Please tell me (1) if there is a book on nursing containing illustrations of instruments used in operations; (2) what book would you recommend on physiology and anatomy for nurses; and (3) is there any book which gives the rules and terms of training for probationers at different hospitals?—*Microbe.*

(1) "Surgical Ward Work and Nursing," by Dr. Miles, contains numbers of such illustrations. (2) You would probably find Dr. Humphry's "Manual of Nursing, Medical and Surgical," would suit your purpose. (3) "How to Become a Nurse" gives these particulars of London and provincial hospitals. All these books can be obtained from the Scientific Press, 28 & 29, Southampton Street, Strand, London, W.C.

*Warm Bed-quilt.*

(44) A few years ago directions were given for making a warm quilt for poor people's beds, in which cotton wool was, I think, used. Can you trace these directions for me?—*M. G. L.*

Perhaps some of our readers can help with suggestions. We believe finely torn-up paper, placed between sheets of some washing material, makes a warm covering.

*Royal Scottish Nursing Institution.*

(45) Can you give me any information respecting the Royal Scottish Nursing Institution, Edinburgh?

We do not know to what institution you refer. The only one we know of bearing the name you give is a private nursing institution at Carlisle. If by any chance you are alluding to the Scottish branch of the Queen's Jubilee Institute you had better write for information to the Lady Superintendent, Miss Wade, 2, Lansdowne Crescent, Edinburgh.

*Children's Nurses.*

(46) We are asked to state with reference to a query under this head last week that ladies are taken for training as children's nurses at Moseley Hall Convalescent Home for Children, Worcestershire.

